# Dysregulated immune cell responses in severe dengue pathogenesis

**DOI:** 10.3389/fimmu.2025.1600999

**Published:** 2025-05-21

**Authors:** Ji-Seung Yoo, Oron Zvi Shporn, Ella H. Sklan

**Affiliations:** ^1^ School of Life Sciences, BK21 FOUR KNU Creative BioResearch Group, Kyungpook National University, Daegu, Republic of Korea; ^2^ Department of Clinical Microbiology and Immunology, Faculty of Medical and Health Sciences, Tel Aviv University, Tel Aviv, Israel; ^3^ The Department of Internal Medicine T, Tel Aviv Medical Center, Tel Aviv, Israel

**Keywords:** dengue virus, severe dengue, dengue fever, immunopathogenesis, immune dysfunction, cytokine production

## Abstract

Life-threatening severe dengue (SD) develops in a small subset of patients suffering from dengue fever (DF), a febrile disease that develops following infection with dengue virus (DENV). DENV is a mosquito-borne positive -sense RNA virus. The rapid spread of DENV vectors, which was exacerbated by climate change and inadequate control measures, has led to outbreaks affecting millions worldwide. There is no specific treatment for DF, and the recently introduced vaccines are ineffective in containing the current outbreaks. Like many other viral diseases, the immune system plays a key role in dengue pathogenesis. The lack of models replicating the disease’s immunopathological features has hampered the understanding of the immune system’s role in developing this disease. Recent advances, such as single-cell approaches, provide better systems and methodologies to study the role of different immune cells in SD, closing this gap and providing a better mechanistic understanding of disease pathogenesis and promoting the identification of targets for therapeutic interventions. Here, we summarize recent advances in SD research, focusing on immune cell interactions and their role in disease severity.

## Introduction

Approximately 1 in 20 patients infected with DENV will develop severe, life-threatening SD. These numbers are substantial, as 100–400 million infections are estimated yearly (1). Mild disease begins abruptly after a 5-7-day incubation period when the patient enters a febrile phase, lasting 2–7 days. Other symptoms might include severe headache, retro-orbital pain, myalgia and arthralgia, rash, and minor hemorrhagic manifestations. Patients deteriorating to SD often have warning signs, including abdominal pain or tenderness, persistent vomiting, clinical fluid accumulation, mucosal bleeding, lethargy, restlessness, and liver enlargement that occur in the late febrile phase around the time of defervescence. The critical phase typically lasts 24–48 hours. While most patients clinically improve during this phase, those with substantial plasma leakage can develop SD. In this stage, systolic blood pressure rapidly declines, and irreversible shock and death may ensue despite fluid resuscitation (1).

DENV’s positive-strand RNA genome is translated as a polyprotein following viral entry via receptor-mediated endocytosis. This polyprotein is then cleaved by viral and host proteases to produce three structural proteins (Capsid, Membrane, and Envelope) and seven non-structural proteins (NS1, NS2A, NS2B, NS3, NS4A, NS4B, and NS5). The viral proteins localize to the endoplasmic reticulum (ER), forming a replication complex built on modified ER membranes (2). The virus replicates from a double-stranded (ds) RNA intermediate transcribed by the viral RNA polymerase. Virus assembly and budding occur at the ER, followed by transport of the virion to the Golgi apparatus, where it matures and proceeds to exit via exocytosis (2).

There are four known DENV serotypes (DEN1-4). Recovery from infection provides lifelong immunity against a particular infecting serotype. However, immunity to the other serotypes is partial. Successive infections by other serotypes increase the risk of SD since the heterologous antibodies from the primary infection are thought to cause antibody-dependent enhancement (ADE). In which pre-existing, non-neutralizing antibodies from prior infections or suboptimal immune responses promote viral uptake into monocytes and macrophages via Fcγ receptors, increasing viral load and the risk for SD (3). While the risk for SD is elevated upon secondary infection, SD also develops during primary infection (4). Furthermore, only a small number of the patients with secondary infection will develop SD. Thus, other factors contribute to the development of SD.

Multiple studies suggest that aberrant immune activation, including cytokine storm, immune cell dysfunction, and impaired interferon response, have a critical role in dengue pathogenesis (summarized in (5–9)). However, it remains unclear why these immune impairments occur in only a subset of infected individuals. Beyond secondary infections, age, genetic predisposition, and comorbidities have been proposed to influence disease severity. Yet, findings on these factors remain inconsistent, suggesting that complex interactions between host immunity and viral mechanisms drive disease progression (10, 11). Here, we focus on the contribution of aberrant immune cell function to dengue pathogenesis, highlighting the gaps in knowledge and potential areas for therapeutic interventions.

## Dendritic cells

Dendritic cells (DCs) are specialized antigen-presenting cells that initiate immune responses by capturing antigens at barrier tissues such as the skin, lung mucosa epithelium, and the nasal and gastrointestinal tract linings (12). DCs uptake antigens primarily through phagocytosis and, upon activation, undergo significant changes. These changes include upregulating surface markers, morphological alterations, and migration to lymph nodes, where they present processed antigens to T cells. Through this process, DCs shape adaptive immune responses by secreting cytokines, chemokines, and costimulatory molecules. Depending on their type and functional state, DCs influence diverse immune outcomes, such as cytotoxic T-cell activation, T-helper cell polarization, and memory B-cell formation (13, 14).

DCs arise from common progenitors, differentiating into classical dendritic cells (cDCs) or plasmacytoid dendritic cells (pDCs). cDCs, which develop in peripheral lymphoid tissues, are highly effective at priming T cells (15, 16). In contrast, pDCs represent a unique subset that produces type I interferons (IFN) (17). During inflammation, circulating monocytes can differentiate into immature monocyte-derived dendritic cells (moDCs), which mature upon stimulation by cytokines or pathogen recognition. These moDCs function predominantly at sites of inflammation, contributing to the local immune response (18, 19).

### DCs are the initial targets of DENV infection

DCs, particularly immature moDCs, are primary targets for DENV infection. DENV infection is mediated by dendritic-cell-specific ICAM3-grabbing non-integrin (DC-SIGN), a C-type lectin expressed on the surface of DCs that recognizes mannose-type sugars on the surface of pathogens to induce phagocytosis. DC-SIGN interacts with carbohydrates on DENV glycoproteins. DC-SIGN levels correlate with susceptibility to infection, with classical and immature moDCs displaying the highest levels (20). Langerhans cells, a specialized subset of skin macrophages in the epidermis, are permissive to infection and disseminate the virus to DCs (21, 22). Following infection, DENV replicates within DCs, producing infectious virions and enabling the migration of infected DCs to lymph nodes, accelerating viral dissemination (23). DENV further enhances monocyte recruitment to the skin, promoting their differentiation into DCs and expanding its cellular targets (24).

### DENV infection impairs DC function

DENV infection disrupts DC functions, particularly maturation and antigen presentation (summarized in (23, 25)). Earlier studies suggested that DENV infection induced DC maturation, as indicated by increased surface markers (26, 27). Later studies, however, distinguished between direct effects on infected DCs and indirect impact on bystander DCs. **Infected DCs** display impaired maturation and altered cytokine secretion. These cells are also more susceptible to apoptosis, limiting their role in stimulating T-cell responses (27–30). However, despite their impaired function, infected DCs retain some crucial functions; for example, infected moDCs can activate natural killer cells (31).

In contrast, **bystander DCs** undergo activation and maturation, driven by inflammatory cytokines such as tumor necrosis factor-alpha (TNF-α) and type I IFNs. They can produce IFN-γ-inducible chemokines when interacting with DENV-specific T cells. This suggests a model in which infected DCs contribute to immune evasion, while bystander DCs help shape the immune response through activation and cytokine signaling (27, 29, 30). This could be a viral immune escape strategy to dampen T-cell responses and impact disease severity.

Notably, exposure to DENV non-structural protein 1 (NS1) can independently stimulate pro-inflammatory cytokine production in moDCs (32). Differences in how DENV serotypes affect DC function, possibly due to varying replication kinetics, highlight the complexity of DENV-host interactions (28). Bystander pDCs were recently reported to interact with infected cells to form a synapse, promoting IFN secretion and a localized antiviral response (33).

### DCs and severe dengue

The impaired function of DCs in infected individuals likely contributes to the progression to SD by disrupting the activation of both innate and adaptive immune responses. However, studies directly linking these mechanisms to SD remain limited. Infection is also thought to cause apoptosis of the infected DCs via several mechanisms, including oxidative stress (25, 30, 34, 35). Apoptosis might also underlie the reduction in the relative proportion of some DC subsets, which was more pronounced in SD patients (36, 37). A reduced abundance of classical dendritic cell type 2 (cDC2s), essential for priming CD4^+^ T cells, was identified in SD progressors compared to patients with DF (38), suggesting impaired adaptive immune responses in severe cases. SD patients also exhibit reduced type I IFN production, which correlates with decreased pDCs, further weakening the antiviral immunity (39, 40).

Additionally, DENV-infected DCs overproduce matrix metalloproteinases (MMP)-9 and MMP-2, which increase endothelial permeability and exacerbate vascular leakage, a hallmark of SD (41). Elevated expression of the Fcγ receptor CD64, associated with ADE, was observed in cDC2 cells in SD progressors, which might exacerbate disease severity (38).

In summary, during DENV infection, DCs are primary targets for the virus, and their infection leads to impaired function, particularly in maturation and antigen presentation, which may contribute to the progression of SD by disrupting both innate and adaptive immune responses.

## Monocytes/macrophages

A common myeloid progenitor differentiates into monocytes and neutrophils in the bone marrow. After their development, monocytes are released into the peripheral blood. Morphologically diverse monocytes comprise approximately 5–10% of human peripheral blood leukocytes (42). After several days, these circulating monocytes differentiate into various tissue-resident macrophages and specialized immune cells, including DCs and osteoclasts.

As key innate immune system components, monocytes and macrophages are integral to host defense, tissue homeostasis, and inflammation. These mononuclear phagocytes recognize and eliminate pathogens and play tissue repair and remodeling roles. Monocyte differentiation is regulated by environmental signals, such as cytokines and inflammatory mediators, driving the formation of macrophage phenotypes with distinct functions. Tissue-resident macrophages respond to local cues, initiating immune responses through phagocytosis, antigen presentation, and the production of cytokines and chemokines (42, 43).

Human monocytes are classified into three subsets based on the expression of CD14 and CD16 surface markers, each with distinct roles in immunity. The most abundant subset, classical monocytes (CD14^++^CD16^-^), accounts for approximately 85% of monocytes in healthy individuals. These cells are primarily involved in phagocytosis, migration, adhesion, and antimicrobial responses. Intermediate monocytes (CD14^++^CD16^+^) share features of classical and non-classical subsets, contributing to antigen presentation, apoptosis regulation, and transendothelial migration. Non-classical monocytes (CD14^+^CD16^++^) play roles in complement and Fc receptor-mediated phagocytosis, vascular homeostasis, and antiviral responses, and they actively participate in transendothelial migration and adhesion. This classification underscores the functional diversity of monocytes and their central role in maintaining immune surveillance, tissue integrity, and inflammation (42, 43). Macrophages are divided into classically activated macrophages (M1) and alternatively activated macrophages (M2). M1 macrophages mediate host defense from pathogens and antitumor immune responses. Meanwhile, M2 macrophages have an anti-inflammatory function and regulate wound healing (44).

### Role of monocytes in viral amplification and dissemination

DENV infects mononuclear phagocyte system cells, including monocytes, macrophages, and dendritic cells, expressing DC-SIGN and mannose receptors, facilitating viral entry (45–49). During secondary infection, ADE further promotes viral uptake into monocytes and macrophages via Fcγ receptors, increasing viral load and the risk for SD (50, 51). While circulating monocytes serve as targets for DENV attachment, they might be less efficient at supporting viral replication than macrophages. These monocytes can act as carriers for viral dissemination and may establish viral reservoirs, exacerbating disease progression (52).

While ADE via Fc receptors is a well-established entry pathway during secondary infection, entry into these cells in primary DENV infections or infections that do not induce ADE occurs through other mechanisms such as the mannose receptor (CD206) and CLEC5A, and TIM-1 and TAM receptors (53–55).

### Monocyte activation and role in severe disease

During DENV infection, monocytes and macrophages undergo significant activation and differentiation, impacting the immune response and disease pathogenesis. Monocytes/macrophages are activated through pattern recognition receptors (PRRs) such as Toll-like receptors (TLRs), retinoic acid-inducible gene (RIG)-I-like receptors, nucleotide-binding oligomerization domain (NOD)-like receptors, and Fc receptor (FcR)-dependent pathways, particularly during ADE (56–59). DENV-infected monocytes express high levels of activation markers like CD32, CD86, and CD11c, in particular, monocytes from SD patients (60). NS1 directly activates mouse macrophages and human peripheral blood mononuclear cells (PBMCs) via TLR4 and induces the production of IL-10 (61, 62). Monocytes can also be activated by apoptotic bodies of DENV-infected cells, further driving inflammation (63). DENV induced activation of monocytes and macrophages leads to the production of cytokines and chemokines, including IP-10, TNF-α, IL-10, IL-6, IL-8, and MCP-1, MIP-1β, IL-1ra (64–66). Monocyte activation and cytokine secretion are associated with increased endothelial damage and vascular permeability, key features of SD (67). Notably, IL-10, a key anti-inflammatory cytokine, may exhibit altered activity in SD, potentially influencing immune cell differentiation and function in a progression- or severity-dependent manner.

DENV infection modulates the number of monocytes, leading to an increase in intermediate monocytes and a decrease in non-classical monocytes. The intermediate monocytes seem to be recruited to draining lymph nodes, which modulate B cells’ response and differentiation (64). Another study suggests that this elevation in intermediate monocytes occurs early during infection but is markedly reduced as the disease progresses (68). A potential role for non-classical monocytes, which are activated following infection and are believed to contribute to hepatic injury in severe cases, has also been suggested (69). In conclusion, dengue infection profoundly impacts the monocyte population by altering their activation state, differentiation pathways, and functional roles. These changes are central to immune defense mechanisms and the pathogenesis of SD.

## Neutrophils

Neutrophils, as key components of the innate immune system, are among the first responders to infection and inflammation. They play critical roles in controlling pathogens through phagocytosis, releasing neutrophil extracellular traps (NETs), degranulation, and producing cytokines and chemokines. Neutrophil-induced NET formation, or NETosis, is a specialized form of neutrophil response in which chromatin and granule proteins are released into the extracellular space to trap and neutralize pathogens. While NETosis plays a key role in antimicrobial defense, excessive or dysregulated NET formation can contribute to inflammation and tissue damage (70). These processes recruit other immune cells, activate antiviral pathways, and help limit viral replication and spread (71). While the impact of DENV infection on dendritic cells and monocytes/macrophages is well-documented due to their role as primary targets of the virus, the contribution of neutrophils to SD pathogenesis remains less well-defined, as it is still unclear if these cells are directly infected and if they support viral replication (72).

Neutropenia has been documented in clinical studies of dengue patients, particularly in pediatric populations. However, the prevalence of neutropenia varies between studies and has not been conclusively linked with severe disease outcomes (73–75). Additional research is needed to resolve these inconsistencies and better understand the relationship between neutrophil dynamics and dengue severity.

### Neutrophil activation and dysfunction in dengue

Neutrophils are activated during dengue infection, as demonstrated by increased expression of activation markers such as CD66b and an enhanced respiratory burst (76). In SD patients, elevated plasma levels of neutrophil elastase and transcriptional markers associated with neutrophil activation and degranulation have been reported (38, 77–79). Additionally, pro-inflammatory cytokines such as IL-8 and TNF-α, known to induce NET formation, are elevated in DF patients, along with evidence of spontaneous nuclear decondensation, an early step in NET production. NETs have antiviral properties, as incubation with DENV-2 reduces viral infectivity (76). Increased levels of NET components have also been detected in the serum of DF and SD patients (76, 80).

DENV infection alters neutrophil differentiation and function. In mouse models, DENV-2 infection led to the generation of immature neutrophils with impaired phagocytic activity and increased NETosis. Promyelocyte HL-60 cells differentiated into dysfunctional neutrophil-like cells upon DENV-2 exposure (81). Infection of purified human neutrophils with DENV2 activates and causes a shift towards altered neutrophils (CD16^bright^/CD62L^dim^ subtype), characterized by delayed apoptosis and diminished phagocytic capacity (82). While secretomes from these DENV-2-stimulated neutrophils reduced viral infection in naive cells, they also induced endothelial cell death, contributing to endothelial barrier dysfunction. Similar phenotypic and functional alterations were observed in naive neutrophils infected with DENV-3, including delayed apoptosis and dysregulated responses (82, 83).

### Neutrophils and severe dengue

While neutrophils can play protective roles by releasing myeloperoxidase, elastase, and NETs, contributing to DENV clearance, excessive neutrophil activation can exacerbate disease severity. Increased levels of myeloperoxidase, elastase, and NET components are associated with vascular leakage and worse outcomes in SD (76, 79, 80, 84). Inhibition of myeloperoxidase in DENV-infected mouse models reduced NETosis and vascular leakage, further highlighting the pathogenic potential of overactive neutrophil responses (81). DENV can also delay neutrophil apoptosis and induce reactive oxygen species (ROS) production, leading to extended inflammation and potential tissue damage (82, 83).

NETs can directly damage endothelial cells through multiple mechanisms involving their structural and enzymatic components. NETs are composed of twenty-four main proteins, including histones, neutrophil elastase, myeloperoxidase (MPO), calprotectin, cathelicidins, defensins, and actin (85). Histones, with their strong positive charge, disrupt cell membranes and lead to endothelial cell death (86, 87). Elastase, a serine protease released during NETosis, degrades extracellular matrix proteins and destabilizes endothelial junctions, contributing to barrier dysfunction (88). Defensins, antimicrobial peptides present in NETs, permeabilize eukaryotic cell membranes and exacerbate endothelial injury (89). Importantly, DNase treatment, which degrades the DNA backbone of NETs, eliminates NET-induced cytotoxicity (90), highlighting the synergistic role of NET structure and associated proteins in endothelial damage.

Beyond pathogen clearance and tissue damage, NETs actively modulate the function of other immune cells through immune crosstalk. PpDCs respond to NETs by producing IFNs essential for antiviral immunity (70). NETs also influence macrophage activation, particularly in atherosclerosis, enhancing cytokine production such as IL-6 and pro-IL-1β. These cytokines promote Th17 cell differentiation and recruit additional myeloid cells, amplifying local inflammation (91). While these processes have not yet been studied in detail in the context of dengue infection, they might occur and contribute to SD.

Thus, neutrophils play a dual role in dengue pathogenesis, contributing to antiviral defense and the development of severe disease. Understanding the balance between protective and pathogenic neutrophil responses is critical for identifying therapeutic targets to mitigate SD complications.

## B cells

B lymphocytes are essential components of the adaptive immune system, primarily responsible for humoral immunity and the formation of immunological memory, which allows for rapid responses to recurring antigen exposures (92).

While B cells are not the primary targets of DENV infection, studies indicate that DENV can infect and replicate in these cells, albeit at lower levels than other immune cells. Infected B cells release only small amounts of infectious virions (93–97). CD300a, a phosphatidylserine receptor, and B cell receptors (BCRs) are thought to facilitate viral entry. Though B-cells express Fc receptors, ADE of DENV infection is not observed in B-cells *in vitro* (93, 98, 99). Instead, a similar mechanism is suggested to enhance B-cell entry via BCR, termed BCR-dependent enhancement (BDE). The accumulation of memory B-cells expressing DENV-specific BCRs after a primary infection makes these cells more susceptible to infection and activation during subsequent infections, thus contributing to the severity of the disease (99). DENV-infected B cells may facilitate viral dissemination, as viral RNA and proteins have been detected in secondary lymphoid organs and germinal centers (45, 49, 100, 101).

### B-cell activation and proliferation

DENV primarily infects naive B-cells expressing IgM/IgD, with the virus found both on the cell surface and intracellularly. Infection triggers increased B-cell activation, as evidenced by upregulated activation markers and altered gene expression (96). Bystander, non-infected B cells also demonstrated immune activation, and IgG1 plasmablasts from two patients exhibited clonal expansion (96). B-cells from dengue patients express higher levels of proliferation markers like Ki-67 compared to controls. Concentrations of B-cell activating factor (BAFF), a cytokine that activates B-cells, are significantly higher in dengue-infected patients. DENV infection induces B-cell differentiation into plasmablasts and plasma cells *in vitro*, a process further enhanced by cytokine stimulation (93). These findings are consistent with reports of increased frequencies of plasmablasts and plasma cells in acute and hospitalized dengue cases (102).

### B-cell contribution to severe disease

B cells produce antibodies that can potentially contribute to ADE, a major risk factor for SD (3). DENV infection has been linked to the production of cross-reactive autoantibodies, such as anti-platelet and anti-endothelial cell antibodies, which may exacerbate disease pathogenesis (103–105). In addition to their antibody-dependent roles, infected B cells produce cytokines and shape the immune landscape in dengue patients. Notably, antibody-independent B cell functions appear to be compromised, potentially due to a reduction in key regulatory subsets such as CD24^hi^CD38^hi^ B cells and CD27^-^ naïve B cells. These subsets, which normally help suppress excessive or pathogenic immune responses, are less responsive to stimulation and fail to produce IL-10 and TNF, likely due to elevated expression of inhibitory Fcγ receptors, including CD32 and LILRB1. This early dysfunction in B cell responses may play a critical role in dengue pathogenesis (106).

The affinity of IgG molecules for different FcγR types is dynamically regulated during immune responses and depends on the Fc domain sequence and Fc-associated glycan composition. SD is associated with an increased abundance of afucosylated IgG1 glycoforms, which have a higher affinity for the activating FcγRIIIa receptor. Higher amounts of afucosylated anti-dengue IgG that develop following primary infection contribute to ADE by increasing the susceptibility to severe inflammation and SD during a subsequent infection. However, despite this contribution, afucosylated IgG cannot explain SD pathogenesis (107).

DENV infection elicits multifaceted responses in B cells, influencing their activation, differentiation, and cytokine production. These changes are critical in the immune response and the pathogenesis of SD.

## T cells

T lymphocytes are critical components of the adaptive immune system, mediating cell-based responses against foreign antigens (108). Upon antigen recognition by the T cell receptor (TCR), naïve T cells undergo activation, clonal expansion, and differentiation into CD4^+^ helper, CD8^+^ cytotoxic effector, and memory T cells. These cells perform diverse immune functions, including direct pathogen elimination, cytokine production, and immune regulation. A small subset develops into memory T cells, providing rapid effector functions upon reencountering the same antigens and, thus, long-term protection (108). A subset of CD4^+^ cells, regulatory T cells (Tregs), play a key role in suppressing the activity of self-reactive immune cells that have escaped central tolerance mechanisms (108).

### T cell activation and differentiation during acute infection

Although DENV does not directly infect T cells, infection significantly impacts T cell dynamics. During the acute phase of DENV infection, there is a notable activation and proliferation of CD4^+^ cells and a massive activation and proliferation of CD8^+^ T cells (109–111). Interestingly, CD4^+^ T cell responses appear to vary with dengue disease severity. Patients with DF show a Th1-type cytokine profile while patients with SD display a Th2-type profile. These observations indicate a severity-dependent CD4^+^ T cell polarization shift during DENV infection (112). Some studies have identified a distinct subset of dengue-specific CD4^+^ T cells that mostly do not show abnormal cytokine expression but display cytotoxic properties. These cells express degranulation markers such as CD107a, mediate cytotoxic activity, and can induce apoptosis in target cells. Together with CD8^+^ T cells, they are thought to play a protective role against SD (110, 113–116).

CD8^+^ T cells respond to primary infection by producing pro-inflammatory cytokines and expressing degranulation markers (117). Their response varies with disease severity: during mild DF, these cells degranulate with reduced cytokine production, while in severe infections, CD8^+^ T cells show an inverse pattern, potentially influencing viral control and disease progression. CD8^+^ T cells can recognize multiple viral determinants and mediate viral clearance by killing infected cells, which may be an essential mediator of protection (117–120).

### T cell dynamics in disease progression

Several studies have indicated that the degree of the T cell response correlates with disease severity. The role of T cells in DENV control and pathogenesis was questioned by other studies that found relatively low circulating T-cells during acute infection, which later increased. One explanation for these small numbers is that skin blister fluid from DENV-infected patients contained a larger number of dengue-specific T cells, suggesting that these T cells may have migrated from the bloodstream to the skin during the infection (121).

During secondary infection, cross-reactive CD8^+^ cells and memory B cells generated during the primary infection can dominate the response, a phenomenon called “original antigenic sin” (122, 123). Contrary to its typical protective role in other infections, “original antigenic sin” can be detrimental during dengue infection. Since these cross-reactive cells and antibodies often have a lower affinity for the virus causing the secondary infection, they can delay viral clearance and lead to higher viral loads. It is proposed that in some patients, a high proportion of T cells skewed towards inflammatory cytokine production without cytotoxicity might contribute to cytokine storm and vascular permeability (110).

T cell exhaustion is a state of reduced immune function, marked by diminished proliferation, cytokine production, and cytotoxicity, often accompanied by high expression of inhibitory receptors like PD-1, LAG-3, and TIM-3. While classical exhaustion is typical in chronic infections, acute dengue can also induce exhaustion-like features, which might contribute to SD. In dengue, T cells, especially γδ, CD4^+,^ and CD8^+^ subsets, show increased PD-1 and TIM-3 expression, sometimes linked to impaired function. However, more research is needed to confirm the link between T cell exhaustion and SD (124–126).

## NK cells

Natural killer (NK) cells are critical components of the innate immune system, playing a pivotal role in the early defense against viral infections, including DENV. They help limit viral spread and initiate the adaptive immune response. NK cells are also regulatory, interacting with DCs, macrophages, T cells, and endothelial cells to modulate immune responses (127).

### NK cells activation and proliferation

NK cells activated early in DENV infection, marked by the upregulation of activation markers such as CD69, CD38, HLA-DR, and adhesion molecules, are thought to associate with mild disease (128). IL-18 is thought to drive their proliferation during the early acute phase of the infection. The responding NK cells have a less mature phenotype and a distinct chemokine-receptor imprint indicative of skin-homing (129).

NK cells kill virus-infected cells through natural cytotoxicity and antibody-dependent cell-mediated cytotoxicity (ADCC). This process involves releasing cytotoxic granules containing TIA-1, perforin, and granzymes, which require cell-to-cell contact and are influenced by type I IFN and TNF-α (130, 131).

NK cells are generally divided into two subsets based on CD56 expression: CD56^high^ and CD56^low^. CD56^high^ cells are primarily cytokine producers involved in inflammation and tissue homing, while CD56^low^ cells are more cytotoxic and crucial for eliminating infected cells. Both subsets are activated during dengue infection, and the balance between them and their activation states significantly impacts the outcome of the infection (132–134).

### NK receptors, ligands, and interactions with dendritic cells

A balance of activating and inhibitory receptors tightly regulates NK cell function. Several activating receptors, including NKp44, NKp46, and NKG2D, are upregulated during DENV infection, with some showing higher activation in cases of DF compared to severe forms (130, 133, 135). In contrast, the inhibitory receptor NKG2A upregulated on activated NK cells may limit NK cell responses during the acute phase of infection (133).

Chemokine receptors are vital for guiding NK cells to DENV-affected tissues, particularly the CD56 ^high^ subset. During infection, responding NK cells express elevated CLA, CCR5, CXCR6, and CCR9 levels, suggesting they are primed to home to peripheral tissues (129).

DENV-infected DCs activate NK cells through direct cell-cell contact mediated by adhesion molecules, triggering NK cell degranulation and IFN-γ production (131, 133). In addition, DCs secrete type I IFNs, TNF-α, IL-12, IL-15, and IL-18, all of which stimulate NK cell responses. This interaction between NK cells and DCs is central to the innate and adaptive immune responses to DENV. NK cells can also kill infected DCs, potentially shaping the subsequent adaptive immune response. Dysregulation in this process may contribute to SD pathogenesis (130, 131, 133).

### NK cells and regulation of disease severity

The activity and phenotype of NK cells are key determinants of disease severity. In the early stages of infection, activated NK cells are associated with milder forms of dengue (128, 130). However, in SD, despite activation, NK cell numbers may decrease, or their cytotoxic potential may be impaired. This reduction in NK cell function may allow for unchecked viral replication and excessive inflammation, leading to a cytokine storm and vascular permeability, particularly in children (130, 133). Genetic factors also play a role in disease outcome, with variations in NK cell receptor genes influencing NK cell function and disease severity (134). Ultimately, the balance between NK cell activation, cytotoxicity, and cytokine production is crucial in determining the clinical course of dengue infection. A robust early NK cell response is linked to milder disease, while an impaired or dysregulated response is associated with more severe outcomes.

## Mast cells

Mast cells (MCs) are immune cells found primarily in the skin and mucous membranes. While MCs are commonly associated with asthma and allergic reactions, they play a critical role as sentinels against pathogens, including DENV, due to their strategic location at sites of potential pathogen entry (136). MCs detect pathogens through pathogen-associated molecular patterns, such as RNA sensors RIG-I and MDA5, and can also respond to indirect infection signals. Upon activation, they initiate antiviral responses by producing chemokines and IFNs, which enhance immune defense and restrict viral replication (137–139). Evidence suggests that MC cell lines and human cord blood-derived mast cells can be directly infected by DENV and produce infectious viruses (140–142).

In the early stages of DENV infection, MCs help limit viral spread. Activated MCs release cytokines like TNF-α and IFN-α, along with chemokines such as CCL5, CXCL12, and CX3CL1, which recruit NK cells and other immune cells to the infection site. These responses facilitate viral clearance and bolster the host’s antiviral defenses (137–139, 143).

### Pathogenic roles of mast cells

Despite their protective functions, MCs can contribute to SD. Upon activation, they release vasoactive mediators—including histamine, tryptase, chymase, serotonin, leukotrienes, and vascular endothelial growth factor (VEGF)—which act on vascular endothelial cells to increase permeability and promote vascular leakage (139, 143–149). Tryptase also degrades fibrinogen and coagulation factors, contributing to coagulopathy (138, 150). DENV-infected MCs further activate endothelial cells via TNF-α secretion, promoting vascular endothelial perturbation and plasma leakage (138, 139, 151, 152). MCs’ inflammatory cytokines and lipid mediators can directly damage the endothelium, compounding vascular complications (151).

Additionally, MCs are susceptible to ADE of DENV infection via FcγRII receptors. This process triggers the release of pro-inflammatory mediators and promotes MC degranulation, amplifying disease severity (139, 140). Serotonin released by MCs can bind to platelet receptors, inducing platelet activation and aggregation and contributing to thrombocytopenia (153). Collectively, these processes highlight the dual role of MCs in dengue: while they are essential for early antiviral defense, their activation during infection can drive vascular leakage, inflammation, and immune dysregulation, exacerbating SD.

## Effects of mosquito saliva on the immune response

Aedes mosquito saliva enhances virus infection through sialokinin, a mosquito-encoded peptide that rapidly reduces endothelial barrier integrity (154). Beyond its effect on vascular permeability, sialokinin also modulates the immune response (155). Studies in humanized mice bitten by sialokinin-knockout mosquitoes revealed increased levels of monocytes and macrophages across multiple organs, along with elevated levels of pDCs, NK cells in the skin and blood, and CD4^+^ T cells. However, these mice exhibited reduced NK T cells in the skin and decreased B cell populations. These findings suggest that the absence of sialokinin promotes a Th1-skewed immune response, implying that under natural conditions, sialokinin likely favors a Th2-dominated response. By suppressing elements of the cell-mediated Th1 response, a critical component in anti-viral defense, sialokinin may contribute to viral pathogenesis (155).

## Progress and challenges in dengue vaccines and antiviral strategies

### Vaccines

Currently, two live attenuated dengue vaccines are licensed: Dengvaxia (CYD-TDV) and Qdenga (TAK-003). Dengvaxia is administered in a three-dose schedule to children and adolescents with confirmed prior dengue infection. It offers approximately 80% protection against symptomatic, virologically confirmed dengue, hospitalization, and SD, with protection lasting at least six years post-vaccination. However, its major limitation is the requirement for pre-vaccination screening, as the vaccine increases the risk of SD in individuals without prior exposure (156, 157). Qdenga, given in two doses, is approved for children aged 6–16 years in regions with high dengue transmission. Its efficacy varies by serotype and prior exposure: it is most effective against DENV-2, moderately effective against DENV-1, and less effective against DENV-3 and DENV-4, particularly in seronegative individuals. Overall efficacy against virologically confirmed dengue ranges from 61% to 82%, with up to 84% protection against hospitalization. Notably, Qdenga induces durable T cell responses and provides protection for up to three years. Its key advantage is that it does not require pre-vaccination screening. However, its lower efficacy against certain serotypes and waning protection over time, especially in younger children, remain challenges (158, 159). Several next-generation dengue vaccines are in advanced clinical development. These candidates aim to provide broader and more durable protection, simplified dosing regimens (e.g., single-dose schedules), expanded age indications, improved safety profiles, and greater accessibility through reduced cost. The wide distribution of such vaccines might significantly lower the burden of dengue infections (160–162).

### Antivirals

The development of effective antiviral therapies for dengue faces several difficulties. First, the existence of four distinct serotypes requires the development of broad-spectrum agents capable of inhibiting all variants. Second, the absence of reliable animal models that accurately replicate human dengue pathology limits preclinical evaluation. Additionally, dengue infection has a narrow therapeutic window: viremia typically peaks early, often before clinical diagnosis, making timely intervention difficult. Finally, as with other RNA viruses, dengue is susceptible to mutations that can lead to antiviral resistance (163–165).

Several candidates have been explored, but progress has been limited. Balapiravir, a nucleoside analog, was discontinued due to a lack of efficacy (166). Mosnodenvir (JNJ-1802), an NS4B-targeting compound, advanced to Phase 2 trials but was later discontinued due to a strategic shift in the manufacturer’s R&D priorities. Subsequently, a viral lineage carrying resistance-associated mutations to this compound was identified (167).

Currently, the only antiviral candidate in clinical development is NITD-688, which also targets the viral NS4B protein. It is undergoing evaluation in Phase 2 trials and remains a promising option for pan-serotype dengue therapy (163–165).

## Translational implications and potential therapeutic targets in SD

The detailed understanding of immune dysregulation in SD pathogenesis, as highlighted in this review, might highlight several promising avenues for immunomodulatory strategies aimed at preventing or mitigating SD.

### Fcγ receptor-mediated enhancement (ADE)

ADE is a risk factor for SD during secondary infections, where pre-existing antibodies facilitate viral entry via Fcγ receptors. Strategies to engineer antibodies with modified Fc domains that retain neutralizing activity without promoting ADE are currently under investigation (168).

### Neutrophil extracellular trap formation

Excessive or dysregulated NETosis has been associated with inflammation and vascular damage in SD. Inhibition of NET formation or mitigation of its downstream effects, such as endothelial cell death, could represent a therapeutic strategy. While some NETosis inhibitors show promise in preclinical studies or are used for other indications, there are currently no therapies that are routinely used in the clinic to inhibit NETosis (90, 169–171).

### Cytokine storm and excessive inflammation

The overproduction of pro-inflammatory cytokines is a hallmark of SD and contributes to vascular permeability. Targeting specific cytokines or their signaling pathways could help to lower this excessive inflammatory response. Understanding the precise roles of key cytokines like TNF-α, IL-1β, IL-6, and IL-8 in SD can assist in the development of therapies aimed at modulating their activity.

### Impaired interferon response

Reduced IFN production in SD is often associated with decreased pDCs levels, suggesting a potential for IFN-based therapies or strategies to enhance endogenous IFN production. While IFN-I has shown efficacy in inhibiting DENV replication *in vitro*, its therapeutic application is less viable if dengue is typically diagnosed after the peak of viremia, a phase when the administration of IFN-I is usually no longer effective.

### Immune cell dysfunction

As highlighted in this review, multiple immune cell populations are central to SD pathology (see also summary in **Table 1**). Identifying the molecular drivers affecting endothelial damage and vascular permeability, such as MMPs secreted by infected DCs or vasoactive mediators released from MCs, could enable the development of therapies targeting these effectors or their cellular sources.

**Table 1:** Summary of immune cell contributions to SD.

Immune Cell type	Contribution to Severe Dengue
Dendritic Cells	Primary targets for infection. Viral dissemination. Impaired function. Impaired cytokine production and activation of the adaptive immune response. Vascular damage.
Monocytes/Macrophages	Targets for infection. Viral dissemination. ADE-mediated infection. Excessive cytokine production. Vascular damage.
Neutrophils	Impaired phagocytic activity. ROS production, extended inflammation, NETosis, endothelial damage.
B Cells	Viral dissemination. Production of cross-reactive autoantibodies. BDE, Produce pro-inflammatory cytokines, increased abundance of afucosylated IgG1.
T Cells	Protective during acute, shift to less degranulation/cytotoxicity in SD and more inflammatory cytokine production. “Original antigenic sin” in secondary infection.
NK Cells	Cytotoxicity is impaired. Impaired dysregulated response. Excessive inflammation. Reduction in function.
Mast Cell	Susceptible to infection. ADE. Release vasoactive mediators- increase endothelial permeability and promote vascular leakage. Excessive inflammation.

Together, these insights into the immune landscape of SD highlight multiple potential therapeutic targets. However, translating these findings into safe, effective treatments remains a significant challenge, underscoring the need for continued research and clinical validation.

## Discussion

**Figure 1:**
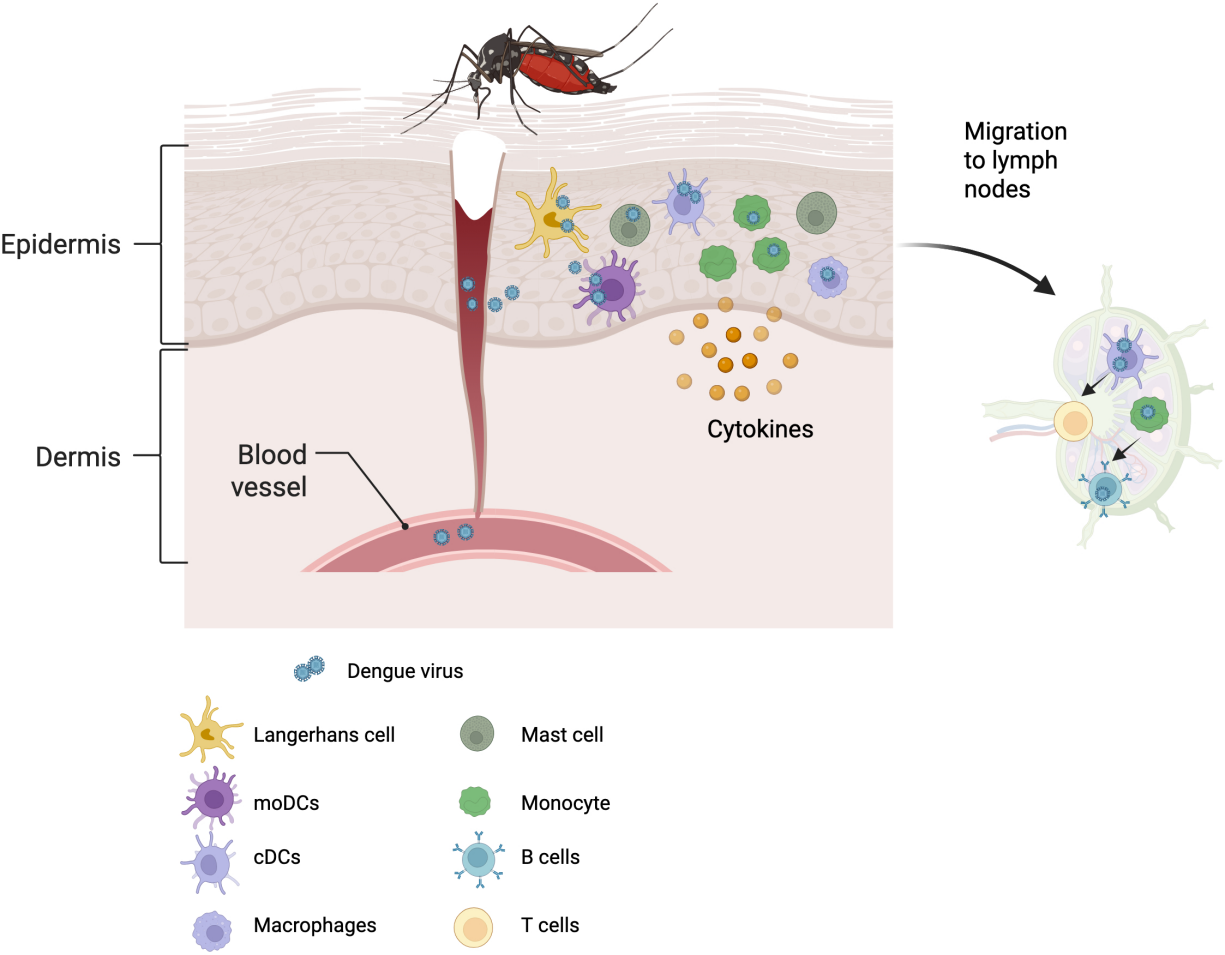
Early immune cell interactions during DENV infection. An infected mosquito transmits the virus into the skin during a blood meal. Langerhans cells, moDCs, and monocytes are the first to encounter the virus, serving as primary replication sites. These infected cells further disseminate the virus by interacting with other immune cells at the site of infection, including other DC subsets, macrophages, and mast cells. The virus reaches microcapillaries directly during the bite or via infected cells that reach the bloodstream. Infected DCs reach peripheral lymph nodes where they attempt to activate T cells by presenting viral antigens. However, their impaired maturation and altered cytokine production (increased IL-10, reduced IL-12 and type I IFN) may lead to a suboptimal and potentially skewed T cell response. Monocytes, including intermediate subsets, also migrate to the lymph nodes and can modulate B cell responses. These interactions activate and shape the adaptive immune response (Created with BioRender.com).

**Figure 2:**
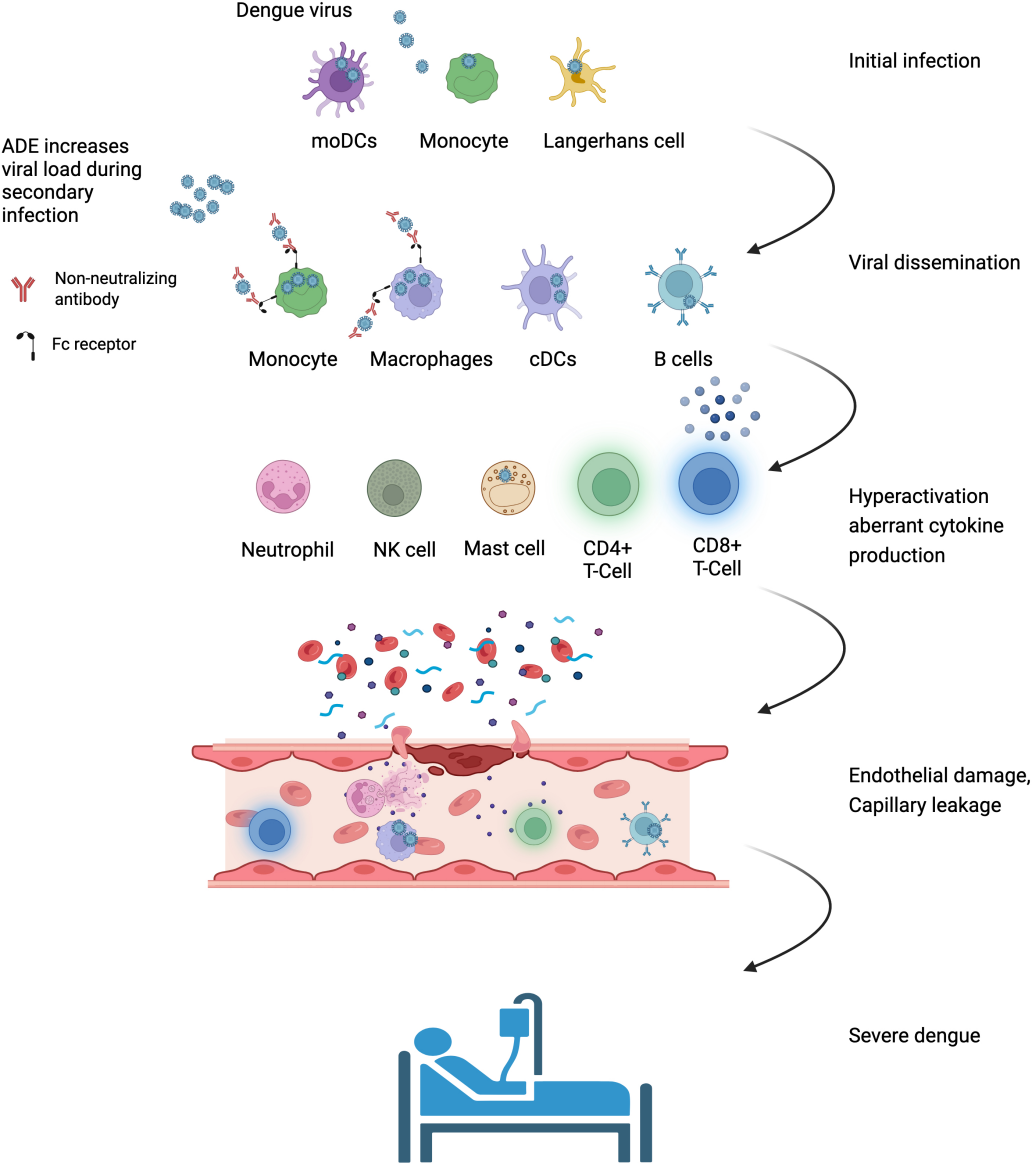
Immune cell contribution to SD progression. Initial infection of immune cells in the skin leads to viral dissemination to the bloodstream and lymph nodes. Mechanisms such as ADE during secondary infection, where non-neutralizing antibodies bind the virus and Fc receptors on monocytes/macrophages, contribute to an increase in viral load. The infection affects multiple immune cell types, leading to altered differentiation patterns and dysregulated cytokine production, impairing viral clearance and causing a further increase in viral load. Excess cytokine production, NETosis, dysregulated vasoactive mediators’ secretion, and additional mechanisms compromise endothelial integrity, contributing to capillary leak and culminating in the development of SD (Created with BioRender.com).

During initial DENV infection, Langerhans cells, moDCs, and monocytes are the first cells to encounter the virus. These cells are the primary targets of viral replication and further disseminate the virus through interactions with other immune cells at the site of infection and in peripheral lymph nodes. **Figure 1** summarizes the events occurring during the initial stages of infection. Local inflammation recruits additional monocytes to the site of infection, further amplifying the infection. The maturation of the infected cells is impaired, affecting functions such as antigen presentation, T-cell priming, cytokine secretion, and other functions, further disrupting the activation of the innate and adaptive immune responses. Some infected cells undergo apoptosis, which diminishes the antiviral response. These effects may initially represent a viral escape strategy to excess viral replication, but they eventually lead to dysregulation and exacerbation of the immune response, leading to SD. While infection affects the infected cells’ function, bystander cells remain active, contributing to the antiviral response. B-cells, cDCs, macrophages, and mast cells are also infected and seem to produce infectious viruses. At the same time, T-cells, neutrophils, and NK cells are also affected, but currently, there is no evidence that the virus replicates in these cells. Although these cells are activated during infection, they produce excess cytokines, undergo NETosis, and display dysregulated secretion of vasoactive mediators. These and additional mechanisms compromise endothelial integrity, contributing to capillary leak and the development of SD. The events leading to SD are summarized in **Figure 2** and **Table 1**. Differences between the effects on infected and bystander cells limited the ability of RNA sequencing and proteomic approaches to identify relevant changes between DF and SD patients using bulk samples of isolated cells. Recent studies using single-cell approaches have identified several key differences between these patient subsets (38, 94).

In agreement with the aforementioned studies, SD progressors displayed differences in immune cell type abundance, gene expression, and molecules secreted compared to DF patients. For example, classical monocytes are expanded and activated in SD patients, possibly promoting migration and conferring sustained inflammation, contributing to SD.

In contrast, nonclassical monocytes are down-regulated, potentially impairing immune surveillance and inducing endothelial damage. Antigen processing and presentation genes are downregulated, and NK cells display reduced cell killing but increased inflammation and migration, further promoting sustained inflammation (38, 94). Additional longitudinal studies on larger, more heterogeneous cohorts of SD patients might highlight specific immune pathways that could help in SD prevention.

A key unresolved question is why only a subset of DENV-infected patients develop these immunopathological features that promote SD. While ADE is a widely proposed mechanism during secondary infections, not all secondary infections lead to SD, and SD can also occur during primary infections (4). Age is another potential factor influencing immune response variability (172, 173). Studies across diverse DENV-infected populations suggest a U-shaped risk curve, where infants and the elderly experience higher SD rates. In infants, an immature immune system combined with maternally acquired antibodies is thought to promote ADE, though not all studies support this (174, 175). In contrast, older individuals in endemic regions face additional risk factors, including an aging immune system, multiple past infections, and comorbidities, which may contribute to SD susceptibility (176). However, some studies report no significant age-related differences in SD incidence among adults (177).

Genetic factors may also influence the immune response and disease severity. Variations in human leukocyte antigen (HLA) alleles and cytokine gene polymorphisms (e.g., TNF-α, IL-10, and IFN-γ) have been linked to SD in several studies (178, 179). However, conflicting findings suggest that population-specific genetic factors and the multifactorial nature of SD contribute to these discrepancies. Understanding the interplay between immune regulation, genetic predisposition, and age-related effects is essential for identifying at-risk individuals and developing targeted interventions for SD.
